# Protective effects of YCHD on the autoimmune hepatitis mice model induced by Ad-CYP2D6 through modulating the Th1/Treg ratio and intestinal flora

**DOI:** 10.3389/fimmu.2024.1488125

**Published:** 2024-11-13

**Authors:** Jiawen Wu, Sixue Lyu, Di Guo, Na Yang, Yang Liu

**Affiliations:** ^1^ College of Basic Medical Sciences, Shanxi University of Chinese Medicine, Jinzhong, China; ^2^ Basic Laboratory of Integrated Traditional Chinese and Western Medicine, Shanxi University of Chinese Medicine, Jinzhong, China; ^3^ Engineering Research Center of Cross Innovation for Chinese Traditional Medicine of Shanxi Province, Jinzhong, China

**Keywords:** autoimmune hepatitis, Yinchenhao decoction, CYP2D6, Th1, Treg, intestinal flora

## Abstract

**Background:**

Autoimmune hepatitis (AIH) is a chronic liver inflammatory disease mediated by autoimmune reactions, the pathogenesis of AIH is probably related to the imbalance of intestinal flora. Yinchenhao decoction (YCHD) has been used to relieve AIH. However, the mechanisms underpinning YCHD’s hepatoprotective effects with the gut microbito have not been fully revealed.

**Objective:**

To explore the potential mechanism of YCHD in treating AIH based on changes in the intestinal flora and Th1/Treg ratio in the spleen and hepatic hilar lymph nodes.

**Methods:**

The AIH mice model induced by the adenovirus vectors that overexpress human cytochrome P450 family 2 subfamily D member 6 (Ad-CYP2D6) was established (untreated group). One week after the Ad-CYP2D6 injection, the AIH model mice were treated by administering YCHD by gavage for 14 days (YCHD-treated group). The therapeutic efficacy of YCHD on AIH was evaluated by detecting the histopathological changes of the liver, serum transaminases (ALT and AST), inflammatory factors (TNF-α,IL-17 and IFN-γ), and autoantibodies (including LKM-1 and LC-1). The ratio of Th1 to Treg within the spleen and hepatic hilar lymph nodes of the mice was detected by flow cytometry. The changes in the species and abundance of intestinal flora and intestinal flora metabolites were analyzed via 16S rRNA gene sequencing and gas chromatography-mass spectrometry (GC/MS) to reveal the protective mechanism of YCHD on liver injury.

**Result:**

YCHD decreased the transaminase activity (AST and ALT), the content of autoantibodies (LC-1 and LKM-1), and the serum TNF-α, IL-12, and IL-17 levels in AIH mice. The degree of inflammatory infiltration in the YCHD-treated group was significantly less than that in the untreated group. YCHD can effectively reverse the abundance and diversity of intestinal flora in AIH mice and affect the release of short-chain fatty acids (SCFAs), especially butyric acid. Moreover, the flow cytometry results showed that YCHD could also decline the ratio of Th1/Treg, which probably be induced by SCFAs via the G protein-coupled receptor (GPR).

**Conclusion:**

YCHD may affect the release of SCFAs by regulating the intestinal microbiota, thereby affecting the differentiation of Th1 and Treg, and achieving the effect of alleviating liver damage.

## 1 Introduction

Autoimmune hepatitis (AIH) is a progressive inflammatory liver disease, characterized by interface hepatitis, lymphocyte infiltration, as well as positive serum transaminase, immunoglobulin G, and serum autoantibodies (1). The incidence of AIH is 0.40/100000 people ~ 2.39/100000 people, and the prevalence rate is 4.8/100000 people ~ 42.9/100000 people (2). At the present stage, the pathogenesis of AIH is still in the exploratory stage, and the risk factors for AIH mainly include the genetic and environmental factors, such as human leukocyte antigen gene, virus, parasite, alcohol, drugs, intestinal flora, etc (3). In addition, gender and age are also vital factors in the onset of AIH with the condition affecting women four times more frequently than men (4–6). The existing treatment regimens mainly rely on non-specific immunosuppression to delay disease progression and prolong survival, but there are still some patients who fail to benefit from the current standard treatment (7).

The intestinal microbiota is a complex and huge micro-ecosystem in the human body and plays a significant role in keeping health by modulating intestinal epithelial development, inducing innate immunity, and regulating human metabolism processes (8, 9). The intestinal flora is a key factor in keeping the balance between intestinal and systemic immune responses. In addition, the intestinal flora metabolites also have a close relationship with immunologic homeostasis. In recent years, an increasing body of research has demonstrated a significant correlation between the occurrence of AIH and disorder within the intestinal microbiota (10, 11). Through 16S rRNA gene sequencing, it was found that the species and abundance of intestinal flora in AIH patients changed significantly, including the increase of *Veillonella*, *Klebsiella*, *Streptococcus*, and *Lactobacillus*, and the decrease of *Clostridium*, *Rumen cocci*, *Vibrio*, *Bacteroides*, and *Fecal cocci* (12). It is indicated that regulating intestinal flora may be a new way to treat AIH.

Yinchenhao decoction (YCHD) was recorded by Shanghanlun in the Han Dynasty of China (150-215 A.D.), which was composed of the *Artemisia caruifolia* Buch, *Gardenia jasminoides* Ellis, and *Rheum officinale* Baill. Modern pharmacological studies also have shown that YCHD has hepatoprotective and cholagogic effects (13). Although YCHD is frequently used to treat liver disorders in clinical practice (14), its hepatoprotective mechanisms are still unclear, especially for the relationship between YCHD and gut microbiota. Therefore, in this study, the Gas Chromatography-Mass Spectrometer (GC/MS) coupled with 16S rRNA gene sequencing techniques were performed to study the effects of YCHD in treating AIH by detecting the changes in the species and abundance of intestinal flora and intestinal flora metabolites, with an AIH mice model induced by the adenovirus vectors that overexpress human cytochrome P450 family 2 subfamily D member 6 (Ad-CYP2D6), to reveal the uncovered intrinsic interaction between YCHD, AIH, and intestinal microflora (15).

## 2 Materials and methods

### 2.1 Experimental animal

24 specific pathogen-free (SPF) male C57BL/6 mice (6-8 weeks old, 18~20g) were supplied by SiPeiFu Co., Ltd. and raised in the Animal Laboratory of Animal Center of Shanxi University of Traditional Chinese Medicine at the conditions of 24.0 ± 2.0°, 55% ± 5% humidity, 12 hours light-dark cycle, free drinking water and diet. All the operations of experimental animals in this study have been approved by the Ethics Committee of Shanxi University of Traditional Chinese Medicine (2019LL41).

### 2.2 YCHD preparation

According to the the research of Shen H et al. (16) and Zhao X et al. (17), YCHD was prepared in the following way: 180g *Artemisia caruifolia* Buch, 120g *Gardenia jasminoides* Ellis, and 60g *Rheum officinale* Baill (all the Chinese medicinal materials were bought from Beijing Tongrentang) were soaked in 1.38L distilled water for 50 minutes and boiled for 30 minutes, the liquid was filtered out. 1.38L distilled water was added to the remaining residue of Chinese medicinal materials and boiled for 30 minutes again. Finally, two parts of the YCHD solution were mixed and concentrated to 50ml.

### 2.3 AIH mice model

Regarding the research of Müller P et al. (18) and Holdener M et al. (19), we selected different concentrations of Ad-CYP2D6 (Shanghai Genechem Co., Ltd.) and different induction times to establish AIH mouse model. Based on the serum transaminase level and the morphological changes in the liver, we finally determined the injection dose of the Ad-CYP2D6 (100μl 1×10^9^ pfu/ml injected via the tail vein) and the time of induction after injection (7 days). The method was shown in **Supplementary Material S1**, and the experimental results were exhibited in **Supplementary Figures S1** and **S2**.

### 2.4 Grouping and drug administration

After a week of adaptive feeding, 24 male C57BL/6 mice were randomly divided into a control group, an untreated group, and a YCHD-treated group (n=8). For the mice in the untreated and YCHD-treated group, 100μl 1×10^9^ pfu/ml Ad-CYP2D6 was injected via the tail vein under sterile conditions. According to the research of Cai FF et al., the mice of the YCHD-treated group were administrated with YCHD on the 7 days after being injected with the Ad-CYP2D6, at the dosage of 10ml/kg per day for14 days (13). The intragastric dose of YCHD was calculated with human and animal body surface coefficient conversion algorithm. At the same time, the mice in both the untreated and control groups were given the same amount of normal saline.

### 2.5 Sample collection

All mice were anesthetized with pentobarbital after fasting for 12 hours. The peripheral blood of mice was collected and centrifuged at 1006 g for 20 minutes at 4°C to obtain the serum. The supernatant was stored at -80°C for biochemical analysis. The liver and spleen were separated and rinsed with normal saline. After being weighed, part of the liver and spleen tissue was immediately placed into the 4% paraformaldehyde solution for histopathology analysis, and the rest was prepared for T-cell subtype analysis with the flow cytometer. The hepatic hilar lymph nodes of mice in each group were collected for Flow cytometry. The small intestine contents were collected in a sterile tube and stored at -80°C for the subsequent detection of intestinal flora and its metabolites.

### 2.6 Liver index and HE stain

The liver index was calculated using the formula: Liver index = liver weight (mg)/body weight (g) to present the liver’s gross morphology change. The histopathological changes of the liver were examined by hematoxylin-eosin (HE) stain with the conventional method, and the necrotic area of liver tissue and the degree of lymphocyte infiltration were observed under a light microscope.

### 2.7 Biochemistry detection

The serum of alanine aminotransferase (ALT) and aspartate aminotransferase (AST) (105-020579-00 and 105-020580-00, Mindray) were detected by BS-240VET Automatic biochemical analyzer for animals (Mindray animal care, China).

### 2.8 ELISA

The contents of serum inflammatory factors, including tumor necrosis factor-α (TNF-α), interferon-γ (INF-γ), and interleukin-17 (IL-17) (MM-0132M1, MM-0182M1, and MM-0170M1, MEIMIAN), and autoantibodies, involving anti-liver-kidney microsomal antibody-1 (LKM-1) and anti-liver cytosol type 1 (LC-1) (MM-47349M1 and MM-46546M1, MEIMIAN), were detected with relevant ELISA kits. All operations were performed according to the kit instructions. Finally, the A51119600 full-wavelength enzyme labeling instrument (Thermo Scientific, USA) was used to determine the absorbance value at the wavelength 450nm.

### 2.9 Flow cytometry

After being removed under an aseptic condition, the spleen and hepatic hilar lymph nodes were washed with normal saline and the excess connective tissue was removed. Then, they were transferred to a metal filter for grinding. The spleen cell suspension was collected and the supernatant was discarded after being centrifuged at 500g for 5 minutes at 4°C. The 3ml red blood cell lysis diluent (10×) was added to the cell pellet for lysing on ice for 3 minutes and then 3ml PBS was added to terminate the lysis. The cell suspension was centrifuged at 500g for 5 minutes at 4°C and the cell concentration was adjusted to 3×10^6^ cells/ml. The hepatic lymph node cell suspension was collected in the centrifuge tube, and the supernatant was discarded after 500g centrifugation for 5 minutes at 4°C and the cell concentration was also adjusted to 3×10^6^ cells/ml. For Th1 detection, 200μl cell suspension was placed in the 96-well plate and incubated with 50ng/ml Phorbol 12-myristate 13-acetate (PMA) (HY-18739, MCE), 1μg/ml Ionomycin (HY-13434, MCE), and 10μg/ml Brefeldin A (BFA) (HY-16592, MCE) at 37° for 5 hours. Cells were blocked with 50μl blocking buffer (2% goat serum, 2% fetal bovine serum, and 96% PBS) for 30 minutes at 4 °, 5μl PerCP Anti-Mouse CD3 Antibody (E-AB-F1013F, Elabscience) and 0.5μl FITC anti-mouse CD4 (B374032, BioLegend) were added for surface staining (incubated at 4°C for 40 minutes). After that, cells were fixed and permeabilized with the eBioscienceT Foxp3/Transcription Factor™ Fixation Buffer (00-5523-00, Thermo), respectively. 50μl blocking solution, 5μl PE anti-mouse CD25 (101904, BioLegend), and 2.5μl APC Rat Anti-Mouse IFN-γ (2010378, BD) were added to perform intracellular staining (incubated at 4°C for 40 minutes). The CD3^+^CD4^+^IFN-γ^+^ Th1 population in the spleen and hepatic lymph nodes was detected by BD Accuric6 flow cytometer.

For Treg detection, 0.5μl FITC anti-mouse CD4 antibody and 5μl PE anti-mouse CD25 antibody were added to 50μl cell suspension for surface staining (incubating at 4°C for 40 minutes) after blocking. The eBioscienceT Foxp3/Transcription Factor™ Fixation Buffer (00-5523-00, Thermo) was added fixing and permeabilization. Another 50μl blocking solution with 5μl Alexa Fluor^®^ 647 anti-mouse FOXP3 (320014, BioLegend) was used for intracellular staining (incubating at 4°C for 40 minutes). Then the CD3^+^CD4^+^CD25^+^Foxp3^+^ Treg population in the spleen and hepatic lymph nodes was also detected by BD Accuric6 flow cytometer. All the data were analyzed using Flowjo10.8.1.

### 2.10 16S rRNA gene sequencing analysis

PCR amplification of the bacterial 16S rRNA genes V3–V4 region was performed by using the forward primer 338F (5’-ACTCCTACGGGAGGCAGCA-3’) and the reverse primer 806R (5’-GGACTACHVGGGTWTCTAAT-3’). Sample-specific 7-bp barcodes were incorporated into the primers for multiplex sequencing. Thermal cycling consisted of initial denaturation at 98°C for 5 minutes, followed by 25 cycles consisting of denaturation at 98°C for 30 seconds, annealing at 53°C for 30 seconds, and extension at 72°C for 45 seconds, with a final extension of 5 minutes at 72°C. PCR amplicons were purified with Vazyme VAHTSTM DNA Clean Beads (Vazyme, Nanjing, China) and quantified by using the Quant-iT PicoGreen dsDNA Assay Kit (Invitrogen, Carlsbad, CA, USA). After the individual quantification step, amplicons were pooled in equal amounts, and pair-end 2×250 bp sequencing was performed using the Illumina NovaSeq platform with NovaSeq 6000 SP Reagent Kit (Shanghai Personal Biotechnology Co., Ltd) (20).

### 2.11 Target metabolites-short chain fatty acid analysis

Extraction of metabolites: The fecal samples of three groups of mice (n=8) were thawed on ice and then put into a 2ml centrifuge tube. 900μl 0.5% phosphoric acid was added to the sample for re-suspension. The mixed liquid was centrifuged at 17949g for 10 minutes. 800μl supernatant was extracted and mixed with the same amount of ethyl acetate. The mixture was stirred for 2 minutes and then centrifuged at 14000g for 10 minutes. 600μl upper organic phase was extracted and mixed with 4-methyl pentanoic acid (the final concentration was 500μM). All the samples were separated by Agilent DB-WAX capillary column (30 m × 0.25 mm ID×0.25 μm). Programmed Heating: Initial temperature of 90°C, then 10°C/min to 120°C, continue to 5°C/min to 150°C, and finally 25°C/min to 250°C for 2 minutes. The Agilent 7890A/5975C gas-mass spectrometer was used for mass spectrometry (See **Supplementary Table S1** for details). The chromatographic peak area and retention time were extracted with MSDChemStation software. The content of short-chain fatty acids (SCFAs) in the sample was calculated according to the standard curve. Finally, the relationship between intestinal flora and SCFAs was comprehensively analyzed on the cloud platform (https://www.genescloud.cn/Home).

### 2.12 Statistical analysis

All values are expressed as the means ± SD using the One-way analysis of variance (ANOVA). The statistical analyses were conducted with SPSS Statistics 27 software, and *P* value < 0.05 was considered as the difference is significant.

## 3 Results

### 3.1 Alleviated liver injury in the YCHD-treated group of mice

**Figure 1:**
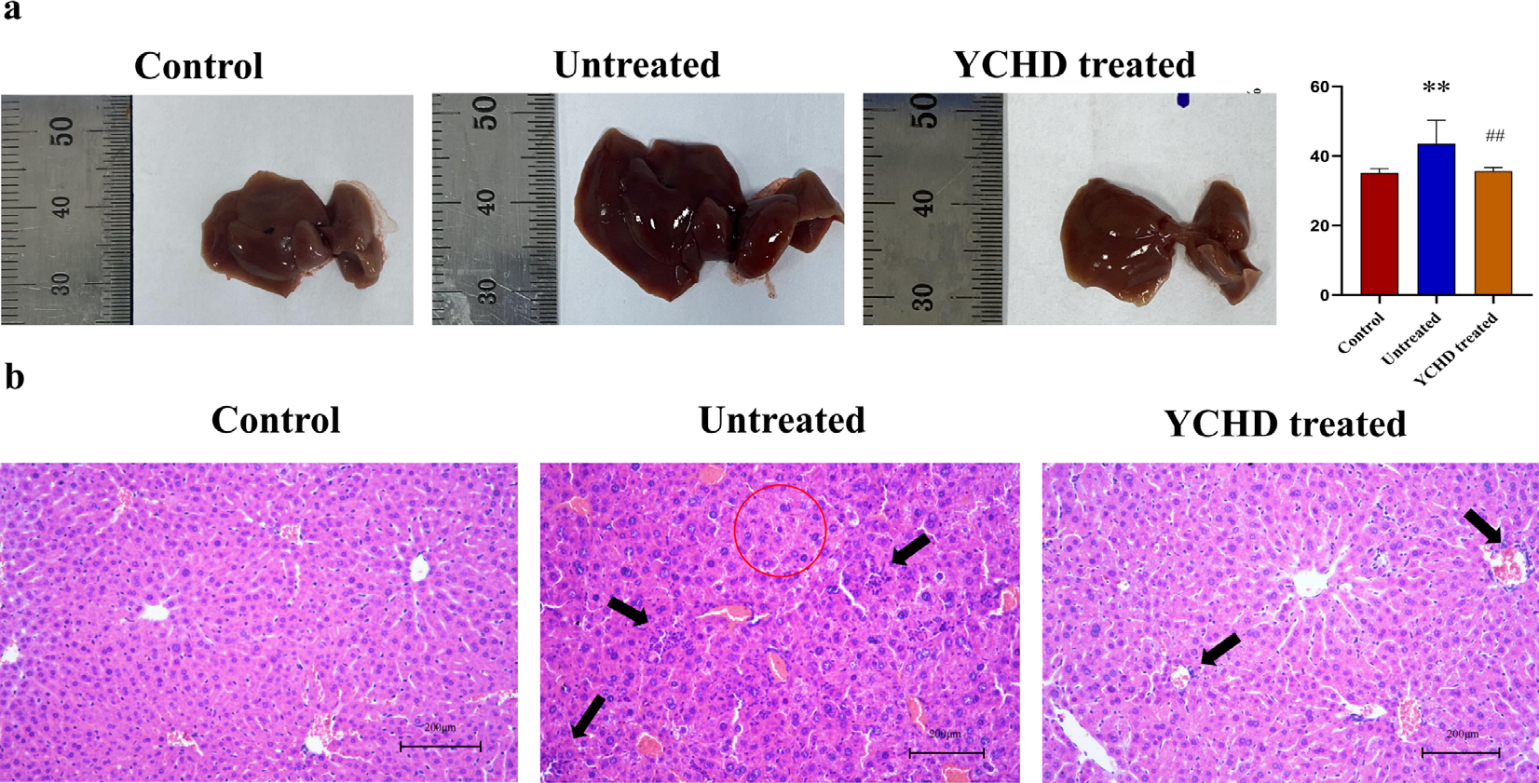
Pathological changes of the liver in each group. **(A)** Gross observation of the liver. The changes in the liver were represented by the liver index. **(B)** HE stain. The red circles are the sites of hepatocyte necrosis, and the black arrows point to the sites of inflammatory infiltrates. Data was expressed as mean ± S.D, n=8. ^**^ *P*<0.01, compared to the control group, ^##^ *P*<0.01, compared to the untreated group.

**Figure 2:**
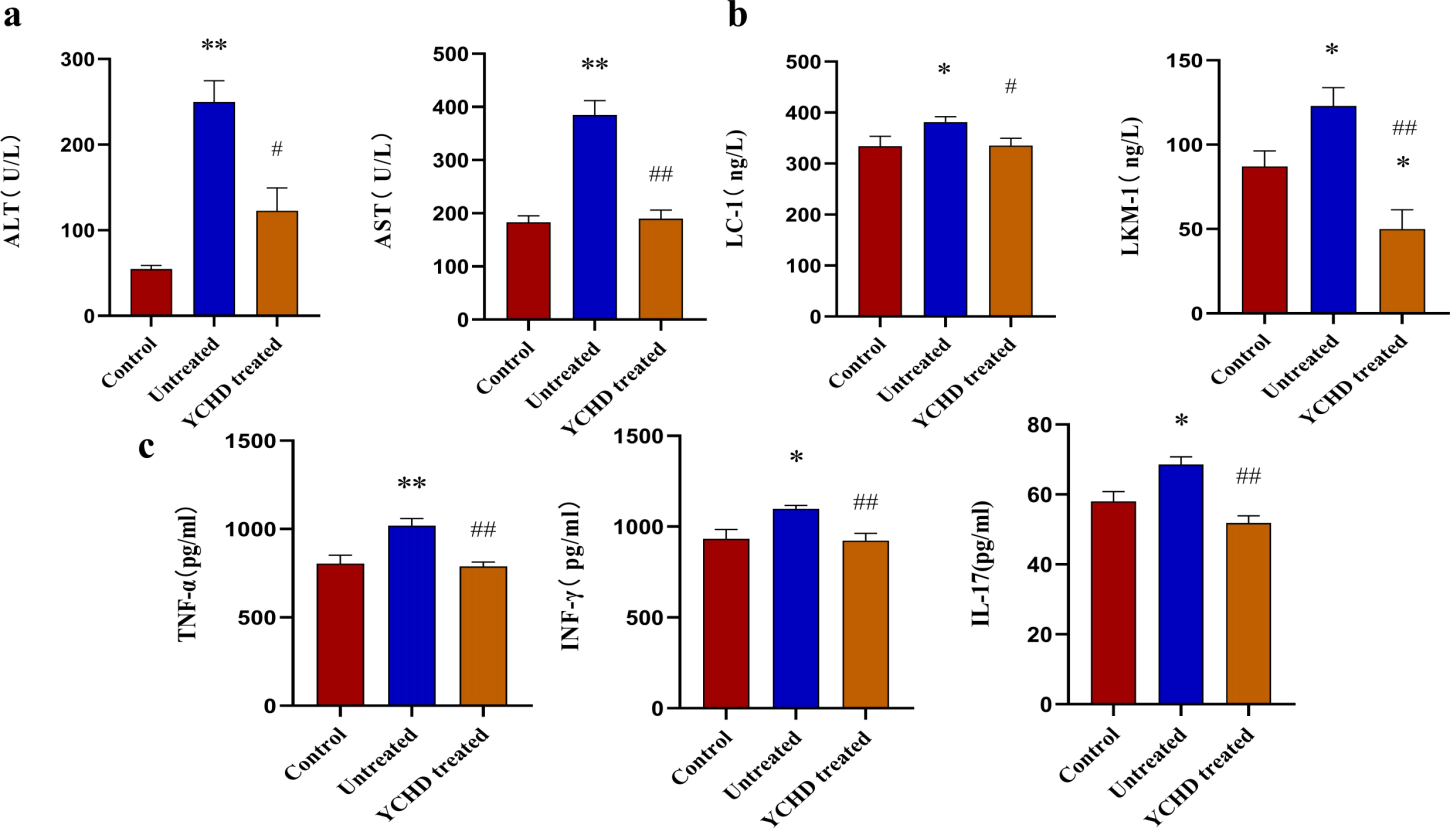
Serum levels of transaminase, autoantibodies, and inflammatory factors detected with the ELISA method. **(A)** Comparison of serum levels of AST and ALT in mice of each group. **(B)** Comparison of serum levels of LC-1 and LKM-1 in mice of each group. **(C)** Comparison of TNF-α, IFN-γ, and IL-17 levels in the serum of mice in each group. Data was expressed as mean ± S.D, n=8. ^*^ *P*<0.01, ^**^ *P*<0.01, compared to the control group; ^#^ *P*<0.05, ^##^ *P*<0.01, compared to the untreated group.

As shown in **Figure 1**, compared with the control group, the liver of mice in the untreated group was enlarged and exhibited a dark red color, and the liver index was higher, however, the liver of mice in the YCHD-treated group was similar to that of the control group, and the liver index was lower than that in the untreated group. The results of HE staining showed inflammatory cell infiltration and massive necrosis of hepatocytes could be found in the untreated group, whereas, the necrotic area and infiltration of inflammatory cells in the YCHD-treated group were alleviated compared to the untreated group. The levels of serum ALT and AST (**Figure 2A**), LC-1 and LKM-1 (**Figure 2B**), IFN-γ, TNF-α, and IL-17 (**Figure 2C**) in the untreated group were significantly higher than those in the control group, whereas, these factors declined in the YCHD-treated group compared to the untreated group.

### 3.2 Declined Th1/Treg ratio in the YCHD-treated group of mice

**Figure 3:**
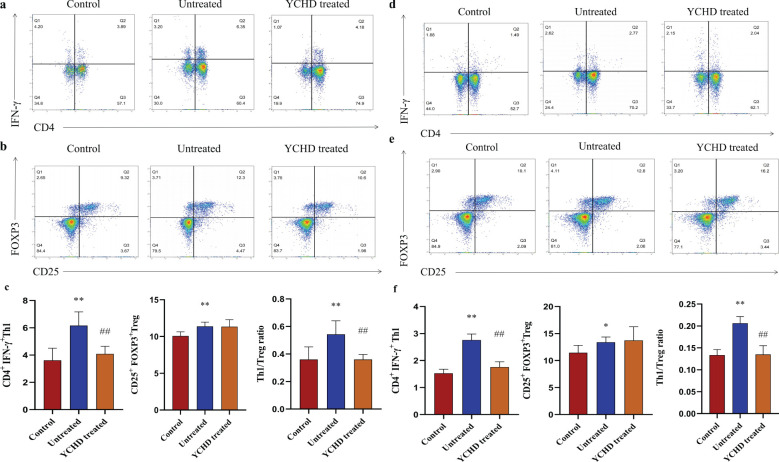
Effect of YCHD on Th1 and Treg cell differentiation. Lymphocytes isolated from the spleen and hepatic lymph nodes of the C57BL/6 mice were stimulated with or without PMA (50ng/ml), ionomycin (1μg/ml), and BFA (10μg/ml) for 5h at 37°. **(A, B)** The gating strategy of Th1 ang Treg cells isolated from the spleen defined as CD3^+^CD4^+^IFN-γ^+^ cells and CD3^+^CD4^+^CD25^+^FOXP3^+^ cells and the representative dot plots from each group are shown. **(C)** Frequency Th1 and Treg cells in the spleen of mice (n=8) in each group were analyzed by flow cytometry. **(D, E)** The Th1 and Treg population in the hepatic hilar lymph nodes was analyzed by staining the cell surface CD4 antigen and IFN-γ antigen, as well as by staining of cell surface CD4 antigen, CD25 antigen, and intracellular Foxp3, in the CD3^+^ T cells population. **(F)** Frequency Th1 and Treg cells in the hepatic hilar lymph nodes of mice (n=8) in each group were also analyzed by flow cytometry. *P<0.05, **P<0.01, compared with the control group. ##P<0.01, compared with the untreated group.

Compared to the control group, the Th1 and Treg population was significantly increased in the spleen of mice, and the ratio of Th1/Treg in the untreated group was higher than that in the control group, while the Th1 population and Th1/Treg ratio in the YCHD-treated group significantly decreased compared to the untreated group (**Figures 3A–C**). In the hepatic hilar lymph nodes, similar changes in the frequency of Th1 and Treg population, and Th1/Treg ratio were found (**Figures 3D–F**).

### 3.3 Reserved gut microbiota dysbiosis in the YCHD-treated group of mice

**Figure 4:**
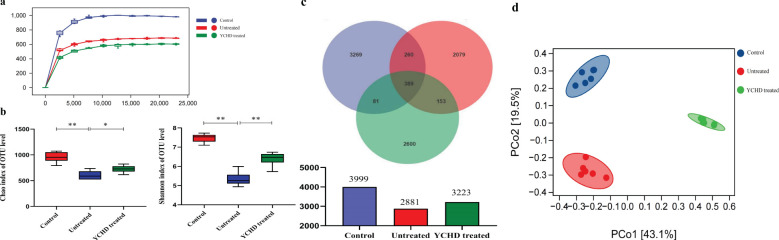
Effect of YCHD on intestinal microflora diversity in AIH mice. **(A)** Sparse curve. The abscissa is the depth of the test (read count), and the ordinate is the numerical value of the diversity index. The dilution curves tend to smooth after reaching a certain value indicating the amount of sequencing data in this study is sufficient. **(B)** The diversity of the gut microbiomes was analyzed with Chao and Shannon indices. The more of the OUT, the more the diversity of the gut microbiomes. **(C)** OTU characteristics among the three groups. Venn plots illustrate the overlap and differentiation of OTUs in the gut microbiota among samples. **(D)** Results of Principal coordinate analysis (PCoA). Significant discriminant taxon nodes of the control, untreated, and YCHD-treated groups are represented by blue, red, and green, respectively. The shorter the distance between samples in the same group, and the more significant the separation between samples in different groups, that indicated the more experimental reliability and rationality of sample selection. Data was expressed as mean ± S.D, n=6. ^**^ *P*<0.01, compared to the control group; ^*^ *P*<0.05, ^**^ *P*<0.01, compared to the untreated group.

The 16S rDNA V3-V4 variable regions of fecal bacteria in 24 samples were sequenced by the Illumina MiSeq platform. The dilution curves of all samples tend to smooth after reaching a certain value (**Figure 4A**), indicating that the amount of sequencing data in this study is sufficient. Based on the Chao1 estimator and Shannon diversity index, YCHD effectively improved the species diversity of AIH mice (**Figure 4B**). According to the Operational Taxonomic Unit (OTU) abundance information, 3999, 2881, and 3223 OTU were identified respectively in the control group, untreated group, and YCHD-treated group through the Venn diagram analysis (**Figure 4C**). Principal coordinate analysis (PCoA) was used to analyze the differences between groups. The points, which represented the data of a mouse’s intestinal flora in different groups, tended to cluster in their respective communities of each group (**Figure 4D**), indicating the characteristic difference of the bacterial community constituted in each group was significant.

### 3.4 Altered gut microbiota composition in the YCHD-treated group of mice

**Figure 5:**
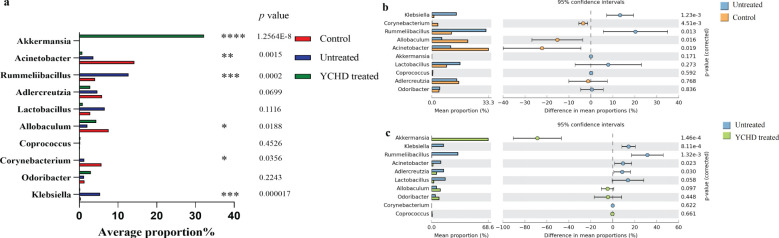
YCHD alters gut microbiota composition in AIH mice. **(A)** Changes in species richness at the genus level between the three groups. **(B)** Changes in species richness between the normal and untreated groups. Bar chart of the Welch T-test at the genus level. **(C)** Changes in species richness between the YCHD-treated groups and untreated groups. Bar chart of the Welch T-test at the genus level. *P<0.05, **P<0.01, ***P<0.001, ****P<0.0001, comparison between control, untreated and YCHD-treated groups.

At the genus level, the top 10 microflora in abundance of all groups were exhibited in **Figure 5A**. Compared to the control group, the relative abundances of *Klebsiella*, *Rummeliibacillus*, *Odoribacter*, *Coprococcus*, and *Lactobacillus* increased in the untreated group, while the relative abundances of *Corynebacterium*, *Acinetobacter*, *Allobaculum*, and *Adlercreutzia* decreased. After being treated with YCHD, the relative abundance of *Klebsiella*, *Rummeliibacillus*, *Adlercreutzia*, *Corynebacterium*, *Acinetobacter*, and *Lactobacillus* decreased in the YCHD-treated group, while the relative abundance of *Odoribacter*, *Coprococcus*, *Allobaculum*, and *Akkermansia* increased (**Figures 5B, C**). The above data suggested that YCHD improved the species composition of the intestinal microbiota of AIH mice.

### 3.5 Altered gut microbiota taxon in the YCHD-treated group of mice

**Figure 6:**
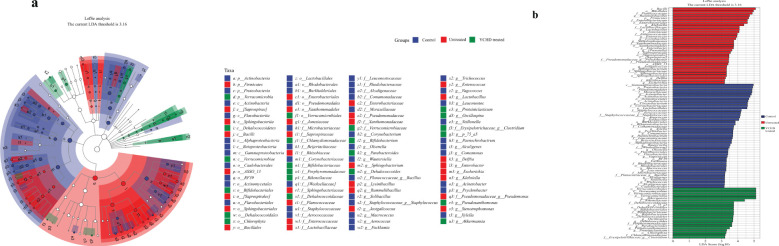
Effects of YCHD on the alterations on taxon of gut microbiota. **(A)** Cladogram of the microbiota. Significant discriminant taxon nodes of the control, untreated, and YCHD-treated groups are represented by blue, red, and green, respectively. **(B)** LDA score of each group. The horizontal bar represented the LDA score, and a threshold value of 3.16 was used as the cutoff level. The horizontal bar chart shows discriminant taxa. Significant discriminant taxons of the control, untreated, and YCHD-treated groups are represented by blue, red, and green, respectively.

To obtain more specific information about the effects of different groups on each taxon, the Linear discriminant analysis Effect Size (LEfSe) and generated linear discriminant analysis (LDA) scores were performed. The LDA value greater than 3.16 was used as the screening criterion to determine the abundance of microorganisms in the group. *Actinobacteria* were more common in the control group, while *Klebsiella* and *Lactobacilliales* were dominant in the AIH group. *Verrucomicrobia* and *Bifidobacterium* accounted for a large proportion of the YCHD-treated group (**Figure 6**).

### 3.6 Elevated SCFA contents in the YCHD-treated group of mice

**Figure 7:**
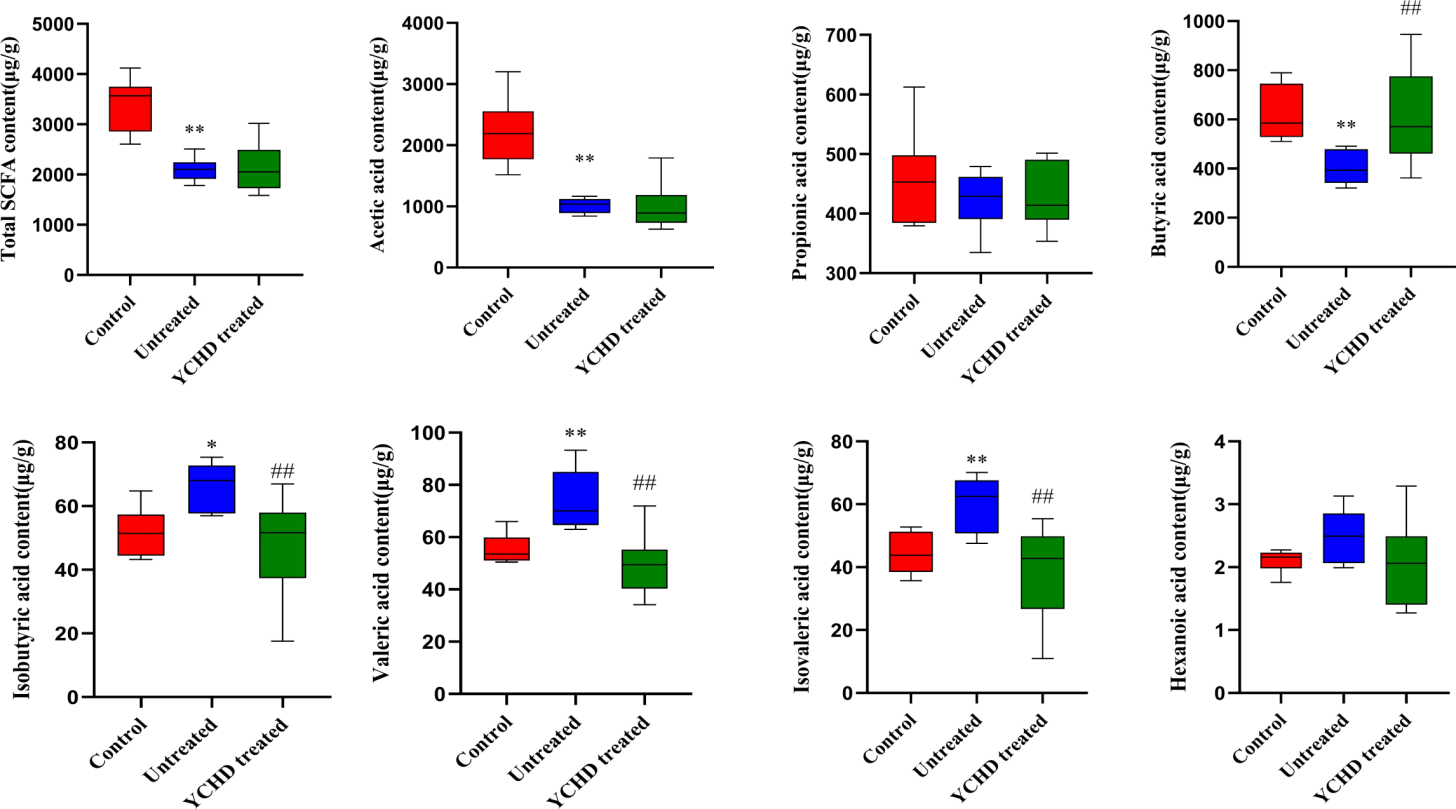
The content of intestinal SCFAs in each group of mice. The control, untreated, and YCHD-treated groups are represented by red, blue, and green, respectively. The horizontal line in the middle of the cabinet is the median of the content of the intestinal SCFAs. ^*^ *P*<0.05, ^**^ *P*<0.01, compared to the normal group; ^#^ *P*<0.05, ^##^ *P*<0.01, compared to the untreated group.

The SCFAs in intestinal contents were detected by the GC-MS method, and the results are listed in **Figure 7**. Overall, the intestinal total SCFA content of AIH mice was significantly lower than that of the control group, while compared to the untreated group, the intestinal total SCFA content of the YCHD-treated group was slightly increased. Compared with the untreated group, the butyric acid and acetic acid content in the YCHD-treated group was increased, however, the difference in acetic acid content between these two groups was not statistically significant. At the same time, the contents of isobutyric acid, valeric acid, and isovaleric acid in the YCHD-treated group were significantly reduced, while the differences in the contents of propionic acid and caproic acid were not statistically significant (See **Supplementary Table S2** for details). Moreover, the Pearson correlation analysis was carried out to reveal the relationship between intestinal microbial composition and SCFA content. The results shown that the abundance of *Corynebacterium* and *Klebsiella* was positively correlated with the content of acetic acid and the content of isobutyric acid, valeric acid, isovaleric acid, respectively. However, the abundance of Klebsiella was negatively correlated with the content of butyric acid (**Supplementary Figure S3**).

## 4 Discussion

The liver and intestines are important parts of the digestive system, engaging in highly complex physiological processes. Under healthy conditions, the intestinal epithelium can protect the body from intestinal microorganisms and their metabolites through a natural barrier including tight junctions, antibacterial molecules, and a mucus layer (21). When the intestinal mucosal barrier is damaged, some pathogenic bacteria will seep out of the intestinal cavity into other organ systems, thus endangering the host’s health. In addition, intestinal flora is involved in metabolism as an important factor in environmental factors, and the disorder of intestinal flora is not only the key factor of intestinal immune response but also an important factor affecting systemic immune response (22, 23). The liver is thought to be the first organ to be exposed to harmful substances in the intestines. Some pathogen-associated molecular models (PAMPs) or metabolites of intestinal flora can enter the hepatic portal circulatory system through the damaged intestinal mucosal barrier, which can cause an inflammatory cascade effect. It will lead to acute injury of hepatocytes and strongly activate the lymphocytes (24, 25). Under certain conditions, it will also stimulate hepatic stellate cells and promote the occurrence and development of hepatic fibrosis (26).

**Figure 8:**
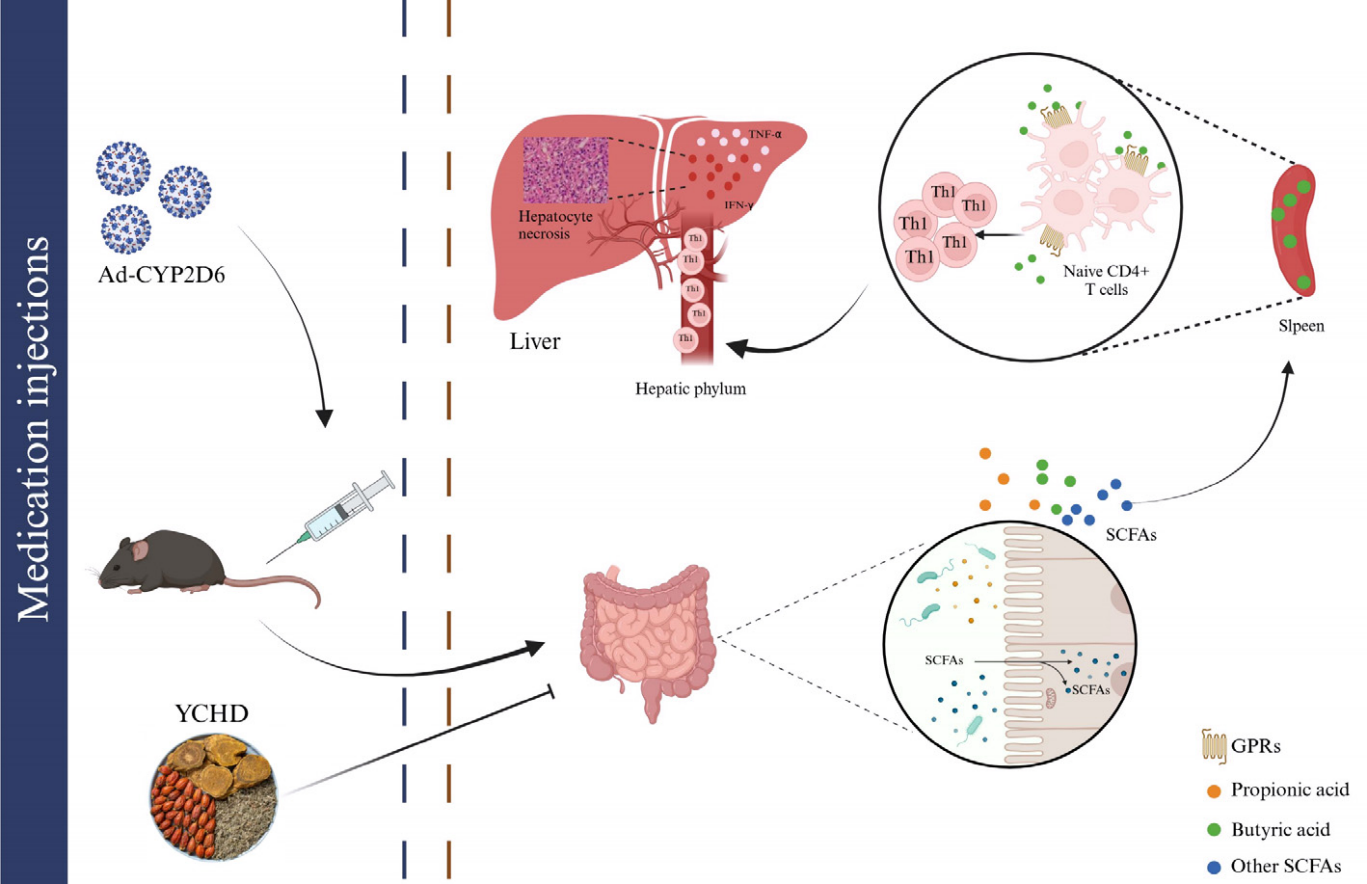
The mechanism of intestinal flora participating in the pathogenesis of AIH by regulating the differentiation of immune cells. Intestinal microbiota disturbances can be observed in AIH mice. Changes in the diversity and richness of species in the gut microbiota can affect the formation of SCFAs, such as butyric acid, propionic acid, and others. The reduction of butyric acid content reduced their inhibitory effect on Th1 differentiation, and the abnormally differentiated Th1 entered the liver through the hilar lymph nodes to induce damage of the liver by releasing inflammatory factors such as TNF-α and IFN-γ. YCHD can treat AIH by blocking the above effects.

Based on the research of Cheng Z et al. (12), the disorder of intestinal flora is closely related to the pathogenesis of AIH. As shown in **Figure 8**, the dysregulation of the microbial region system leads to the disruption of the intestinal barrier, which results in some intestinal microorganisms transferring from the intestine to the liver. Some of these intestinal microorganisms can be regarded as a continuous reservoir of antigens. After the intestinal barrier is damaged, the intestinal flora can continue to present antigens, which may disrupt the dynamic balance of immunity homeostasis in the liver and result in the continuous occurrence of autoimmune reactions. Some studies have shown that the intestinal microbial composition of patients with AIH changed significantly, the abundance of *Clostridium*, *Rumen cocci*, *Vibrio*, *Bacteroides*, and *fecal cocci* declined, while the abundance of *Veillonella*, *Klebsiella*, *Streptococcus*, and *Lactobacillus* elevated (22). The diversity and total load of the intestinal flora of the new HLA^-^DR3^+^ mouse model immunized with human CYP2D6 were significantly lower than that of healthy mice (27). All these prove that intestinal flora may be involved in the pathogenesis of AIH, and suggest that regulating the homeostasis of intestinal flora may be a new strategy for treating AIH.

The balance of Treg cells and Th1 cells plays an important role in maintaining immune homeostasis and is closely related to the occurrence and development of numerous autoimmune diseases. AIH is characterized by Th1-biased immune responses, and Th1 cells, responsible for TNF-α and IFN-γ, have been reported to be highly represented in the liver inflammatory infiltrates of AIH patients (28, 29). Studies have shown that the occurrence of AIH may be related to defects in the number and function of Treg cells. The immunosuppressive function of Treg cells is mainly dependent on the continuous expression of the transcription factor forkhead box protein 3 (Foxp3) and the production of immunosuppressive cytokines including IL-10 and TGF-β (30). Alterations in the Th1/Treg balance may be responsible for AIH progression. However, the regulatory mechanism of T cell subset differentiation in the development of AIH is not fully understood.

YCHD is the representative Chinese herbal compound prescription for treating AIH in ancient and modern Chinese medicine clinical practice. However, it is still difficult to fully explain the pharmacodynamic mechanism of the liver-protecting effect for YCHD at present. In this study, except for confirming its hepatoprotective effect by detecting the morphological and serological changes (including transaminase, inflammatory cytokine, and autoantibody), we found that YCHD can reverse the ratio of Th1/Treg in the spleen of the AIH model mice (**Figure 3**). Moreover, YCHD can also regulate the species diversity of intestinal flora of the AIH model mice based on the diversity indexes, including the Chao1 estimator and Shannon diversity index (**Figure 4B**). According to previous studies, the depletion of beneficial bacteria and expansion of potential pathobionts are associated with AIH disease status (25). Therefore, we also analyzed the effects of YCHD on the composition of intestinal microflora at the genus level. According to the results exhibited in **Figure 5**, it was indicated that *Klebsiella*, *Rummeliibacillus*, *Lactobacillus*, *Allobaculum*, especially *Akkermansia* may have a close relationship with the hepatoprotective activity of YCHD because the abundance of these gut flora was significantly altered compared to the untreated group after intragastric administration of this herbal compound. It has been reported that *Akkermansia* is a new probiotic that plays a protective role in improving liver disease, and it may maintain the intestinal barrier and inhibit inflammation by regulating the release of SCFAs that affect the differentiation of Treg cells, or affecting other types of signaling pathways to protect the liver (31–34).

At the same time, we also found that YCHD can influence the production of intestinal flora metabolites, such as SCFAs. Based on the results of GC-MS analysis listed in **Figure 7**, the contents of isobutyric acid, valeric acid, and isovaleric acid were decreased, while the contents of acetic acid, butyric acid, and propionic acid were increased in the YCHD-treated group. It has been found that SCFAs, especially butyric acid, serve as key messengers facilitating communication between the intestinal microbiome and the immune system (12). SCFAs bind histone deacetylase (HDAC) or G protein-coupled receptor (GPR) signals, including GPR41, GPR43, and GPR109A expressed in the intestinal epithelium and immune cells, to modulate the intestinal epithelial barrier function as well as maintaining the homeostasis of mucosal and systemic immunity (35–38). GPR41 exists widely in a variety of tissues, while GPR43 is mainly expressed in lymphoid tissues and various immune cells. GPR41 and GPR43 can bind to acetate, propionic acid, and butyric acid, while GPR109a can be activated by butyric acid. Since SCFAs can enter the blood circulation, they also have a wider range of systemic effects. An increase in Foxp3^+^Tregs was observed in mice given SCFAs (39). Moreover, SCFAs can also influence the differentiation of naive T cells into effector T cell subsets, such as helper Th1 and Th17 cells. SCFAs can promote the microbiota’s antigen-specific IL-10 production in Th1 cells through GPR43 and induce the differentiation of Th1 and Th17 cells upon exposure to immunological challenges. Butyric acid is one of the representative SCFAs, and we have found that YCHD can affect butyric acid production in AIH model mice in this study. Butyric acid is absorbed mainly by the intestinal epithelium. The anti-inflammatory effect of butyrate is achieved by directly affecting the differentiation of intestinal epithelial cells, phagocytes, B cells, and plasma cells as well as Tregs and effector T cells (40). It can induce the activation of NLRP3 inflammatory bodies and the secretion of IL-18 by colonic epithelial cells, promote the recruitment of neutrophils to inflammatory sites, and enhance the differentiation of Foxp3^+^ Tregs, which can then inhibit the activation of effector T cells (41–43). Most important of all, it is found that butyric acid can not only promote the differentiation of Treg but also have a certain effect on the differentiation of Th1. For instance, butyrate induces differentiation of intestinal Treg cells by promoting histone H3 acetylation of Foxp3 promoter and other conserved non-coding region Foxp3 genes (39). Butyrate also accelerates the oxidation of fatty acids by converting to the downstream activation of butyl-CoA and carnitine palmitoyltransferase 1A. It has been shown that this up-regulation of fatty acid oxidation promotes the differentiation of Treg *in vitro* (43). And, it has been reported that butyric acid can promote Tregs amplification and increase the expression of IL-10 by GPR109A-expressed macrophages and dendritic cells (44, 45). At the same time, it has also been proved that butyrate upregulates the expression of IL-10 in Th1 cells isolated from healthy individuals and IBD individuals through the GPR43-mediated transcription factor BLIMP1 (46). Meanwhile, excessive concentrations of butyrate and butyric acid can promote the differentiation of Th1 cells by up-regulating T-bet expression and down-regulating the expression of RORγt and other Th17 cell-related transcription factors (47, 48). In this study, we found that the effect of butyric acid on Treg differentiation may be higher than that of Th1 differentiation. However, the role of butyric acid in the differentiation of different lymphocytes still needs to be further studied. Moreover, some studies have shown that propionic acid can directly inhibit the production of IL-17 by γδ T cells through an HDAC-dependent mechanism, and inhibit the secretion of IL-17 in human γδ T cells from patients with IBD (49). Although we found the change in propionic acid content could play a positive role in protecting the liver injury, it still needs to be further investigated. To sum up, it was indicated that YCHD may affect the content of SCFAs (including butyric acid and propionic acid) by regulating the abundance and diversity of intestinal flora and then regulating the immune homeostasis of the body by reversing the ratio of Th1/Tregs. This is summarized in **Figure 8**.

By mass spectrometry, the main active components of YCHD include 7-methoxycoumarin, quercetin, Rhein, Genipin, and emodin-8-β-D glucoside (the experimental results are published separately) (50). According to related studies, quercetin may be a key ingredient in regulating intestinal flora and immune response by YCHD (51–53). Quercetin is a kind of natural flavonoid widely found in flowers, leaves, and fruits of plants, which is mostly in the form of glycosides (54). A large number of studies have shown that quercetin has anti-inflammatory, antioxidant, anti-allergic, anti-virus, and other biological activities (55, 56). Studies have shown that polyphenols can regulate intestinal microorganisms, and quercetin may be one of the main components (57–60). Yu et al. reported that quercetin significantly changed the composition of intestinal flora in Wistar rats (61). At the phylum level, the relative abundance of *Chlamydia* and *Cyanobacteria* increased significantly, while the relative abundance of *Bacteroides* decreased. At the genus level, the relative abundance of *Candidatus Arthromitus*, *Lactococcus*, *Bacillus subtilis* and *Rumencocci* which are beneficial to host health increased significantly. Gwiazdowska et al. showed that the inhibitory effect of quercetin on *Bifidobacterium* cultured *in vitro* was dose-dependent and with the strength of inhibition intensifying as the dose increases (62). Therefore, quercetin in YCHD may be an important substance for its hepatoprotective and immunomodulatory effects through the “intestine-liver” axis. However, the above mechanism needs to be verified by further experiments.

## 5 Conclusion

In this study, we found that YCHD has a positive effect on liver injury in the Ad-CYP2D6-induced AIH mice model. Its therapeutic mechanism may be related to regulating the abundance and diversity of intestinal flora of mice, which will subsequently affect the release of SCFAs, such as butyric acid. SCFAs will bind to the GPR receptor on the surface of T cells, thereby affecting T cell differentiation, and ultimately achieving the effect of alleviating liver damage.

## Data Availability

The original contributions presented in the study are publicly available. This data can be found here: NCBI repository, accession number PRJNA1172145.

## Supplementary material

Supplementary Material S1 The specific method of model building. Regarding the study of Müller P et al. (18), we selected 100μl different concentrations (0.5×10^9^ pfu/ml, 1×10^9^ pfu/ml, and 2×10^9^ pfu/ml) of Ad-CYP2D6 to establish the AIH mice model via tail intravenous injection under sterile conditions. 7 days after intravenous administration, compared with the control group, the content of serum transaminase and the degree of infiltration of inflammatory cells in liver tissue were not significantly changed in the 0.5×10^9^ pfu/ml group but significantly changed in the 1×10^9^ pfu/ml and 2×10^9^ pfu/ml group. However, the mortality in the 2×10^9^ pfu/ml group was higher than that in the 1×10^9^ pfu/ml group (shown in **Supplementary Figure S1**). Therefore, 100μl 1×10^9^ pfu/ml was finally determined as the optimal dosage of Ad-CYP2D6 to establish AIH mice for the next experiment. According to the research of Müller P et al. (18) and Holdener M et al. (19), we also explored the induction time of the mouse model. On the 3, 7, and 14 days after the Ad-CYP2D6 injection (100μl 1×10^9^ pfu/ml), the content of serum transaminase and the morphological changes in the liver of the mice were detected. The results showed that on the 3 days of Ad-CYP2D6 injection, the aminotransferase levels of the mice did not show significant changes, and no obvious inflammatory cell infiltration or liver tissue necrosis were found under microscopic observation. However, on the 7 and 14 days after Ad-CYP2D6 injection, the aminotransferase levels of the mice were significantly increased, and a large number of inflammatory factors infiltrated and some hepatocytes necrosis were observed under the microscope. Since there was no significant difference in liver function between the mice on day 7 and day 14, we chose day 7 after Ad-CYP2D6 injection as the time point for model success (shown in **Supplementary Figure S2**).

Supplementary Figure 1 Effect of different Ad-CYP2D6 concentrations on establishing the AIH mice model. **(A)** The mortality of mice in different concentration of Ad-CYP2D6 group. **(B)** Comparison of serum levels of AST and ALT in mice of low, medium, and high concentrations. **(C)** HE stain. The black arrows point to the sites of inflammatory infiltrates. Data was expressed as mean ± S.D, n=5. ^**^ *P*<0.01, compared to the control group.

Supplementary Figure 2 Effect of different induction times after Ad-CYP2D6 injection on establishing the AIH mice model. **(A)** Comparison of serum levels of AST and ALT in mice of 3 days, 7 days and 14 days after Ad-CYP2D6 injection. **(B)** HE stain. The black arrows point to the sites of inflammatory infiltrates. Data was expressed as mean ± S.D, n=5. ^*^ *P*<0.05, compared to the control group, ^#^ *P>*0.05, compared to the 7 day.

Supplementary Figure 3 Pearson correlation analysis between the contents of SCFAs and the abundance of intestinal microbiota in each group. Each box represented the correlation between different samples. The red box indicates positive correlation and the blue box represents negative correlation. The redder or bluer the box, the higher the relevance.

Supplementary Table 1 Specific temperature descriptions used for mass spectrometry.

Supplementary Table 2 Content of specific SCFA in different group.
