# Supplementary material for: Protective effects of YCHD on the autoimmune hepatitis mice model induced by Ad-CYP2D6 through modulating the Th1/Treg ratio and intestinal flora
Source: Front Immunol. 2024 Nov 13;15:1488125. doi: 10.3389/fimmu.2024.1488125 (PMC11600021; doi:10.3389/fimmu.2024.1488125)
Supplement: Supplementary Material S1 — The specific method of model building. Regarding the study of Müller P et al. (18), we selected 100μl different concentrations (0.5×10^9^ pfu/ml, 1×10^9^ pfu/ml, and 2×10^9^ pfu/ml) of Ad-CYP2D6 to establish the AIH mice model via tail intravenous injection under sterile conditions. 7 days after intravenous administration, compared with the control group, the content of serum transaminase and the degree of infiltration of inflammatory cells in liver tissue were not significantly changed in the 0.5×10^9^ pfu/ml group but significantly changed in the 1×10^9^ pfu/ml and 2×10^9^ pfu/ml group. However, the mortality in the 2×10^9^ pfu/ml group was higher than that in the 1×10^9^ pfu/ml group (shown in Supplementary Figure S1 ). Therefore, 100μl 1×10^9^ pfu/ml was finally determined as the optimal dosage of Ad-CYP2D6 to establish AIH mice for the next experiment. According to the research of Müller P et al. (18) and Holdener M et al. (19), we also explored the induction time of the mouse model. On the 3, 7, and 14 days after the Ad-CYP2D6 injection (100μl 1×10^9^ pfu/ml), the content of serum transaminase and the morphological changes in the liver of the mice were detected. The results showed that on the 3 days of Ad-CYP2D6 injection, the aminotransferase levels of the mice did not show significant changes, and no obvious inflammatory cell infiltration or liver tissue necrosis were found under microscopic observation. However, on the 7 and 14 days after Ad-CYP2D6 injection, the aminotransferase levels of the mice were significantly increased, and a large number of inflammatory factors infiltrated and some hepatocytes necrosis were observed under the microscope. Since there was no significant difference in liver function between the mice on day 7 and day 14, we chose day 7 after Ad-CYP2D6 injection as the time point for model success (shown in Supplementary Figure S2 ). [file Table1.docx]

**Supplementary table 1.** Specific temperature descriptions used for mass spectrometry.

| Ion source temperature | 230℃ |
| --- | --- |
| Transmission line temperature | 250℃ |
| Quadrupole temperature | 150℃ |
| Inlet temperature | 250℃ |
