# Supplementary figures and images for: Protective effects of YCHD on the autoimmune hepatitis mice model induced by Ad-CYP2D6 through modulating the Th1/Treg ratio and intestinal flora
Source: Front Immunol. 2024 Nov 13;15:1488125. doi: 10.3389/fimmu.2024.1488125 (PMC11600021; doi:10.3389/fimmu.2024.1488125)

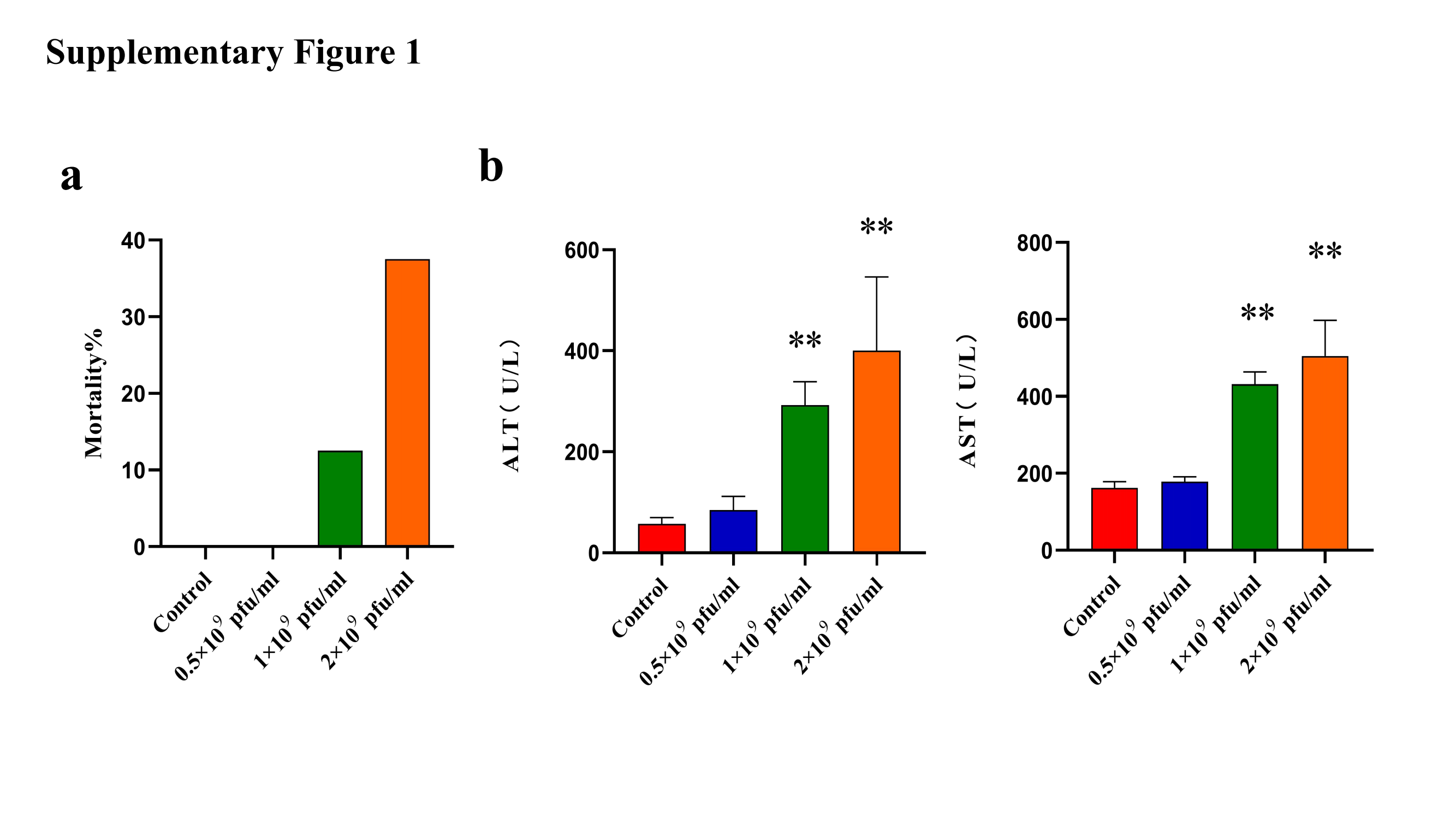

Supplement: Supplementary Figure 1 — Effect of different Ad-CYP2D6 concentrations on establishing the AIH mice model. (A) The mortality of mice in different concentration of Ad-CYP2D6 group. (B) Comparison of serum levels of AST and ALT in mice of low, medium, and high concentrations. (C) HE stain. The black arrows point to the sites of inflammatory infiltrates. Data was expressed as mean ± S.D, n=5. ** P<0.01, compared to the control group. [file Image1.tif]

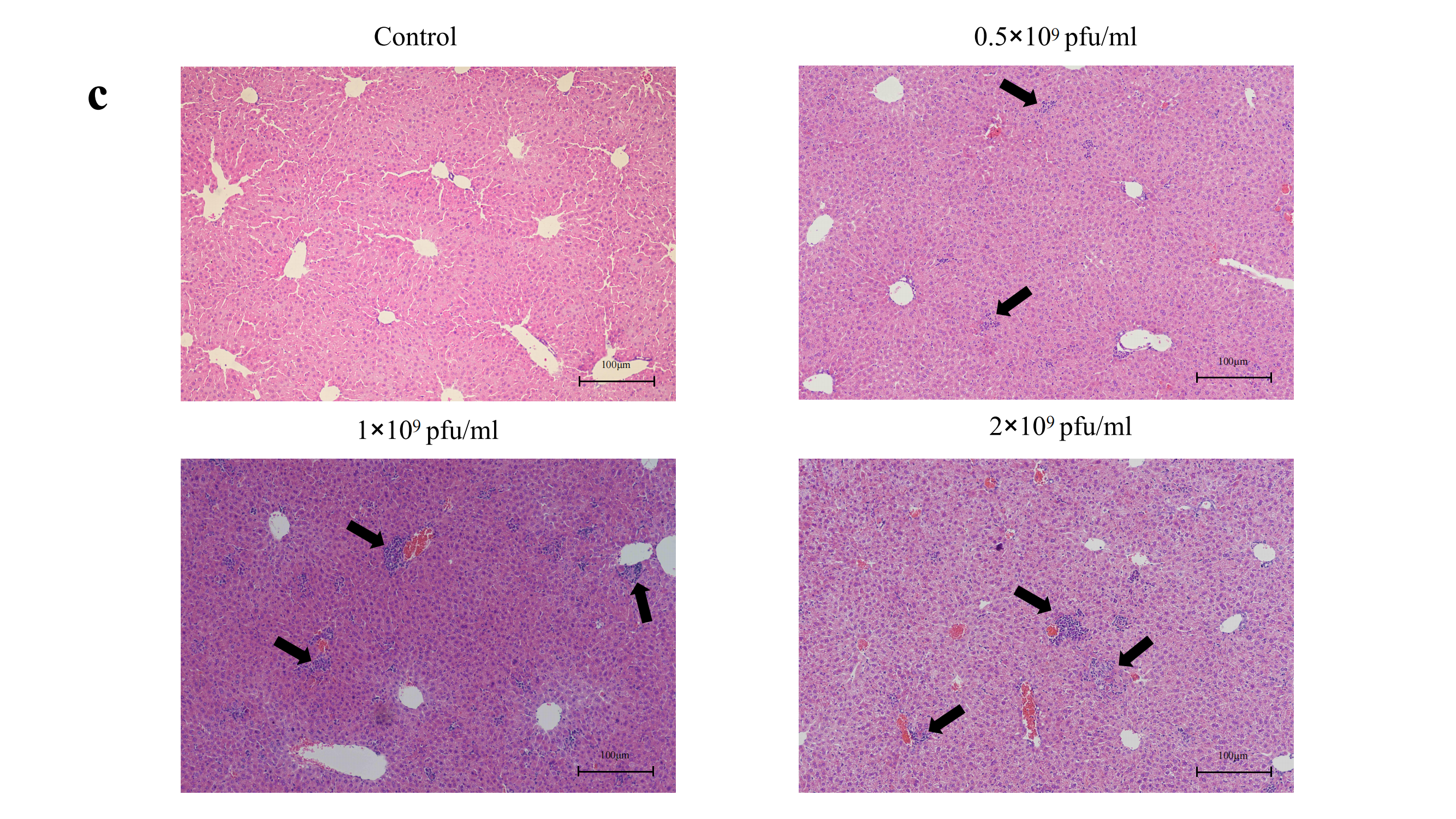

Supplement: Supplementary Figure 2 — Effect of different induction times after Ad-CYP2D6 injection on establishing the AIH mice model. (A) Comparison of serum levels of AST and ALT in mice of 3 days, 7 days and 14 days after Ad-CYP2D6 injection. (B) HE stain. The black arrows point to the sites of inflammatory infiltrates. Data was expressed as mean ± S.D, n=5. * P<0.05, compared to the control group, # P>0.05, compared to the 7 day. [file Image2.tif]

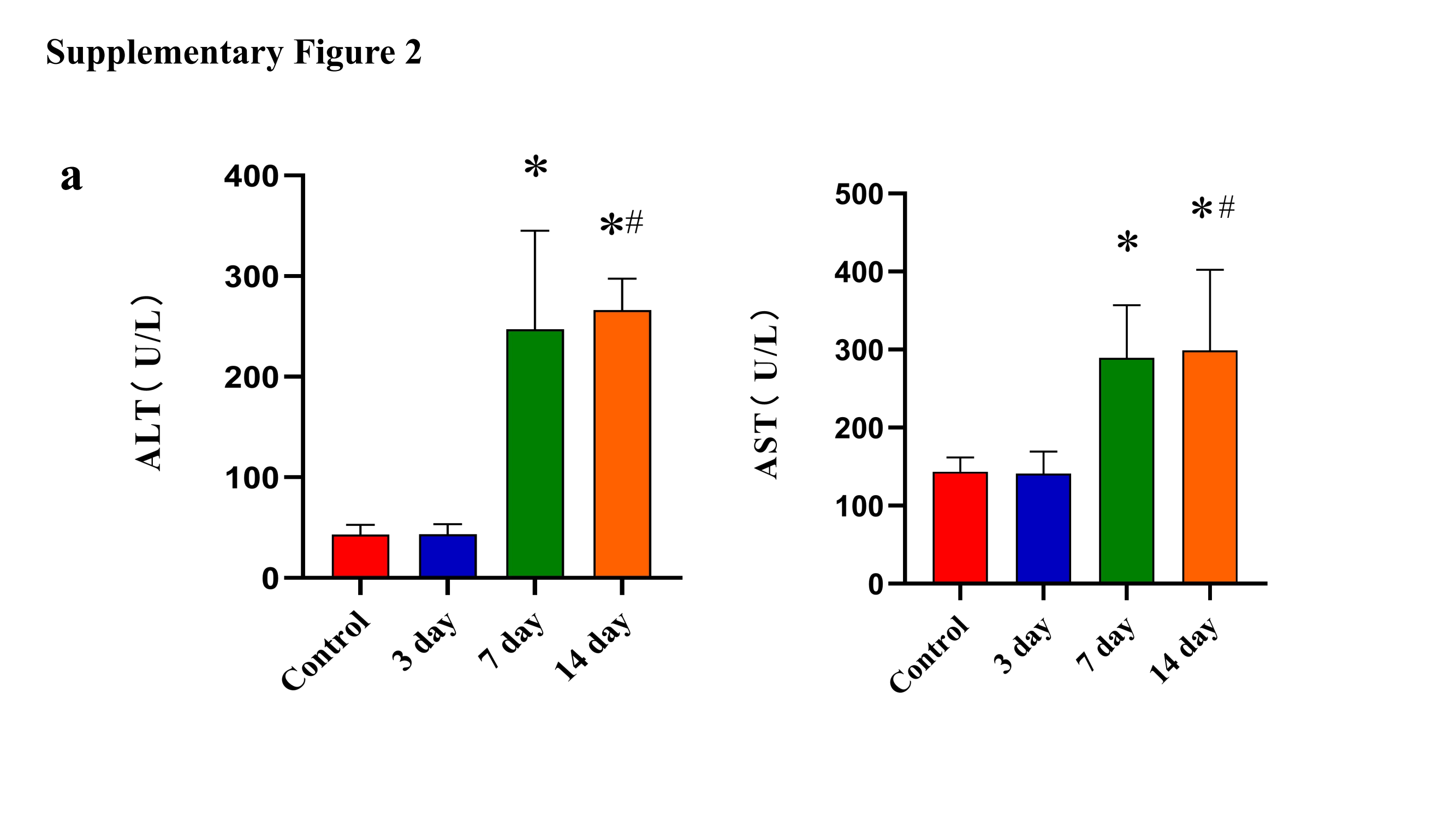

Supplement: Supplementary Figure 3 — Pearson correlation analysis between the contents of SCFAs and the abundance of intestinal microbiota in each group. Each box represented the correlation between different samples. The red box indicates positive correlation and the blue box represents negative correlation. The redder or bluer the box, the higher the relevance. [file Image3.tif]

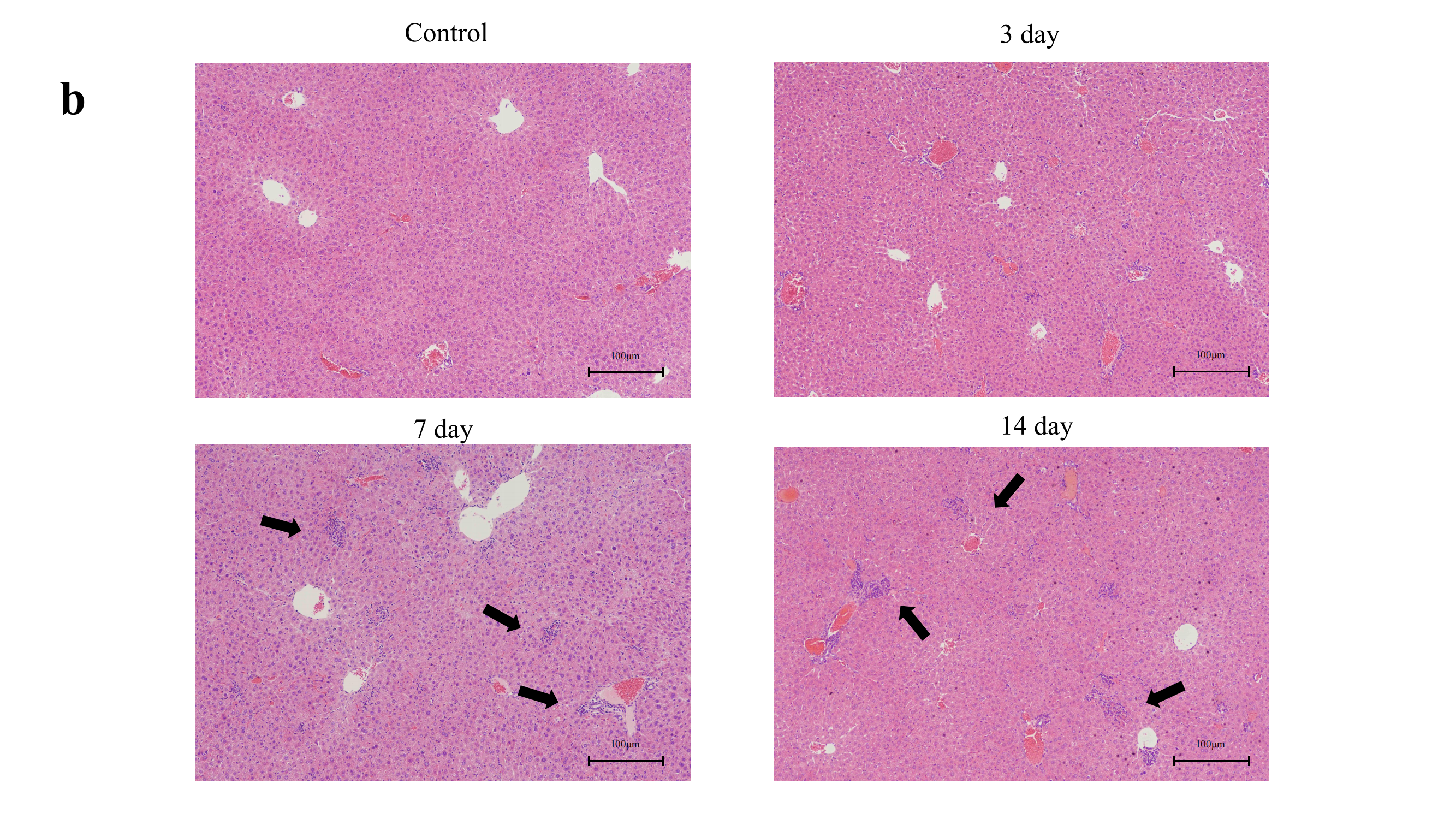

Supplement: Supplementary file 5 [file Image4.tif]

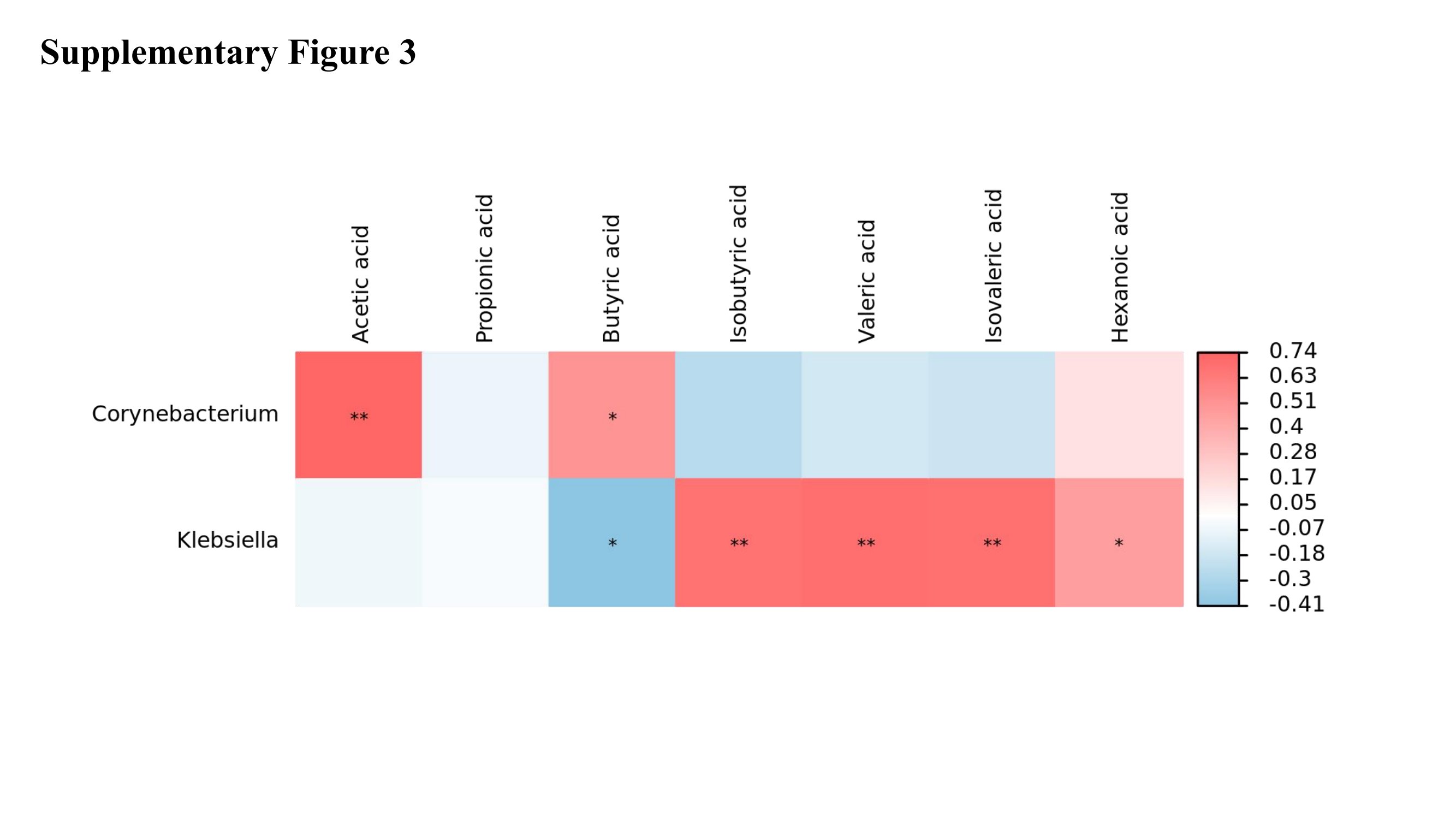

Supplement: Supplementary file 6 [file Image5.tif]
