# Supplementary material for: Protective effects of YCHD on the autoimmune hepatitis mice model induced by Ad-CYP2D6 through modulating the Th1/Treg ratio and intestinal flora
Source: Front Immunol. 2024 Nov 13;15:1488125. doi: 10.3389/fimmu.2024.1488125 (PMC11600021; doi:10.3389/fimmu.2024.1488125)
Supplement: Supplementary Table 2 — Content of specific SCFA in different group. [file Table2.docx]

**Supplementary table 2.** Content of specific SCFA in different group.

| Group  SCFAs | Control | Untreated | YCHD-treated |
| --- | --- | --- | --- |
| Total SCFA (μg/g) | 33.89±5.27% | 21.02±2.26% | 21.33±5.04% |
| Acetic acid (μg/g) | 22.01±5.36% | 10.19±1.18% | 10.04±3.77% |
| Propionic acid (μg/g) | 4.56±0.78% | 4.22±0.47% | 4.30±0.55% |
| Butyric acid (μg/g) | 6.21±1.08% | 4.04±0.66% | 6.09±1.92% |
| Isobutyric acid (μg/g) | 0.51±0.07% | 0.66±0.07% | 0.48±0.15% |
| Valeric acid (μg/g) | 0.55±0.05% | 0.74±0.11% | 0.49±0.11% |
| Isovaleric acid (μg/g) | 0.44±0.06% | 0.59±0.08% | 0.38±0.14% |
| Hexanoic acid (μg/g) | 0.02±0.001% | 0.02±0.004% | 0.02±0.006% |
